# Toward multiplex health: integrating complexity, normativity, and Open Science

**DOI:** 10.3389/fpsyg.2025.1603474

**Published:** 2025-09-08

**Authors:** Junaid Qadir, Diana Maddah, M. Walid Qoronfleh, Recep Senturk

**Affiliations:** ^1^College of Engineering, Qatar University, Doha, Qatar; ^2^College of Health Sciences, Qatar University, Doha, Qatar; ^3^Healthcare Research & Policy Division, Q3 Research Institute (QRI), Ypsilanti, MI, United States; ^4^College of Islamic Sciences, Hamad bin Khalifa University, Doha, Qatar

**Keywords:** multiplex health, critical complexity, systems thinking, BioFiqh, Open Science, semiotics, comparative ethics, precision health

## Abstract

Contemporary healthcare remains constrained by models grounded in linear causality, predictive logic, and biomedical reductionism—models that often fail to address the lived, relational, and spiritual dimensions of health, especially under uncertainty. This paper introduces the Multiplex Health (MH) framework as a coherent alternative, rooted in critical complexity theory and multiplex ontology and epistemology. MH advances six core principles: (1) *multiplex ontology*—viewing humans as multi-layered beings encompassing material, metaphysical, and spiritual domains; (2) *multiplex epistemology*—integrating empirical, experiential, and interpretive ways of knowing; (3) *pluralistic modeling*—combining mechanistic, statistical, and semiotic approaches; (4) *critical complexity*—recognizing health as emergent, open, and irreducible to single models; (5) *triangulated science*—linking Big Data and Small Data, and balancing prediction with understanding; and (6) *comparative multiplex ethics*— drawing on Islamic *BioFiqh* to integrate legal, moral, and spiritual reasoning in health decision-making. By foregrounding the behavioral, ethical, and conceptual dimensions often overlooked in conventional approaches, MH offers a foundational framework for advancing Population Health Management (PHM). A PHM case study focused on mental health illustrates how MH can navigate complexity, enhance relational care, and broaden the scope of well-being beyond reductionist paradigms. MH challenges the dominance of closed, optimization-driven models in precision health and artificial intelligence, instead calling for a “both-and” logic that embraces uncertainty, diversity, and contextual nuance.

## 1 Rethinking health in complex systems

The biomedical model, dominant in modern healthcare systems, is increasingly recognized as insufficient for addressing the full complexity of human health. Rooted in Enlightenment-era science and Cartesian dualism, it privileges linear causality, biological mechanisms, and predictive logic (Engel, 1977, 1992). This reductionist approach has delivered remarkable biomedical advances but continues to struggle with chronic illness, mental health, and the subjective dimensions of healing. As George Engel (1977) argued in his seminal critique, medicine must expand beyond its 17th-century worldview to include psychological, social, and existential dimensions.

Critiques of this uniplex model have emerged across decades and disciplines. Capra (1984) and Capra and Luisi (2014) emphasized the failure of mechanistic thinking to account for the dynamic, interdependent nature of life systems. Sturmberg and Martin (2013) assembled a comprehensive critique of the limitations of Evidence-Based Medicine (EBM), showing how it often fails to meet the needs of real-world clinical practice in complex adaptive systems. Sturmberg (2009) further critiques EBM as an overly narrow methodology that ignores context and subjective experience and becomes fixated on standardized interventions while neglecting the uniqueness of each patient’s journey.

The concept of health itself demands rethinking. As Sturmberg (2012) notes, the word “health” is derived from the Old English *hal*, meaning “whole.” Health, in this view, is not merely the absence of disease but the experience of wholeness. The WHO (1946) foundational definition of health as “a state of complete physical, mental and social well-being” also gestures toward this expansive view, though its operationalization often remains biomedical.

This narrowing of how we define and know health has far-reaching consequences for emerging technologies like machine learning (ML) and predictive artificial intelligence (AI). These tools often operate under the assumption that health can be fully understood through patterns in past data, treating it as a closed system—one that is fully observable, predictable, and governed by stable regularities. Proponents of “scientific wellness” argue that data-driven, personalized approaches can revolutionize prediction and prevention (Hood et al., 2004; Hood and Price, 2023). Yet despite their promise, these models often rely on the same reductionist assumptions. As we elaborate later in the paper, such Closed ML approaches (Birhane and Sumpter, 2022) tend to abstract away contextual nuance, relational dynamics, and lived experiences—and can be especially limited or even harmful when applied to individuals at the social margins, where complexity and structural inequities are most pronounced.

Taleb (2012) offers a sharp critique of the healthcare system’s emphasis on optimization and control, arguing that such approaches cultivate fragility in complex systems. In response, he introduces the concept of antifragility—the ability of a system to thrive and improve under conditions of variability, stress, and uncertainty—as a more suitable objective than mere predictability or stability. Taleb also revives the term *iatrogenics* (from Greek *iatros*, physician, and *genesis*, origin), highlighting harm from medical intervention. This aligns with Illich’s (1976) warning against overmedicalization and the erosion of self-care. Similarly, Vogt et al. (2016) caution that precision medicine may pathologize normal variation, leading to unnecessary treatments. In contrast, the MH framework promotes resilience by minimizing naïve intervention, emphasizing lifestyle and preventive care, and empowering individuals to take ownership of their well-being.

As Birhane (2021) argues, such models struggle with context, fail to account for interpersonal dynamics, and risk reinforcing inequalities. Birhane and Sumpter (2022) advocate for a shift toward “critical complexity,” which recognizes that real-world health systems are open, adaptive, and not fully predictable. Narayanan and Kapoor similarly warn that predictive AI can overfit historical patterns, falter when conditions change, and mislead decision-making by ignoring its own systemic impacts.

Moreover, health cannot be separated from meaning. Senturk et al. (2020), Birhane (2021), and Juarrero (1999) emphasize that in complex, open systems, understanding must go beyond causal explanation to include interpretive frameworks. Health is a deeply personal and subjective phenomenon shaped not only by biological mechanisms but also by symbols, relationships, and cultural narratives—layers that predictive logic alone cannot fully capture (Sturmberg, 2012).

In response to these limitations, we propose the Multiplex Health (MH) framework. MH is grounded in critical complexity theory and embraces a plural, integrative, and layered understanding of health. It synthesizes both explanatory and interpretive modes of knowing, incorporating diverse epistemologies, including spiritual and ethical traditions. MH affirms that health is emergent, relational, and value-laden, and that decision-making under uncertainty must integrate meaning-making, context, and ethics.

## 2 The MH framework: multiplex ontology, epistemology, and methodology

The MH framework is based on a philosophical worldview based on the Multiplexity framework proposed by Senturk et al. (2020) to address the complex realities of human health. In this section, we discuss multiplex ontology, epistemology, and methodology.

### 2.1 Multiplex ontology

**Figure 1:**
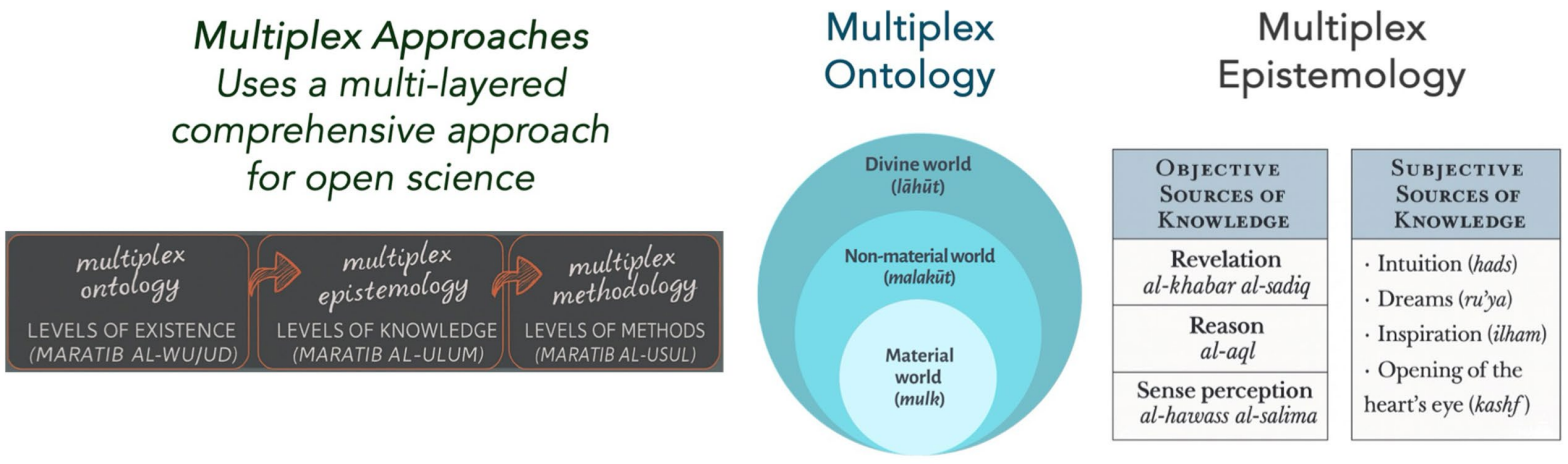
The Multiplex Health (MH) Framework, rooted in traditional Islamic metaphysics, presents a layered ontology—material (mulk), spiritual (malakūt), and divine (lāhūt)—and embraces diverse knowledge sources: empirical, rational, and intuitive. Unlike reductionist uniplex models, MH supports an open scientific paradigm that addresses the full human reality—body, soul, and spirit. Adapted from Senturk et al. (2020).

The MH framework rejects the reductionist ontology underlying most biomedical paradigms, drawing instead from a multiplex understanding of human nature rooted in Islamic metaphysics and complexity science. Senturk’s (2022, 2025) *marātib al-wujūd* (hierarchies of existence) outline a layered ontology comprising the material body, psychological self, and spiritual soul. These layers are not arranged in a rigid hierarchy of value or dominance but are understood as integrative dimensions of reality, each reflecting a distinct yet interdependent facet of human experience (Figure 1).

The Qur’anic verse, *“We will show them Our signs in the horizons (āfāq) and in themselves (anfus) until it becomes clear to them that this is the truth”* (Qur’an 41:53), captures this layered semiotic ontology. Health is understood as a convergence of external order (environmental and social context) and internal equilibrium (spiritual and emotional integrity). The body is not merely a biomechanical system but a sign-bearing entity whose well-being must be interpreted in relation to divine, moral, and ecological balance.

While MH draws inspiration from traditions that recognize a metaphysical layering of reality—such as the Islamic view that situates the body, self, and soul within a hierarchy—it does not impose a rigid epistemological dominance. By focusing on shared moral, relational, and existential dimensions of health, it can be meaningfully contextualized for secular and non-theistic worldviews. This approach stands in contrast to dominant modern scientific paradigms that deny the transcendent and operate under the illusion of metaphysical neutrality— but effectively abolish metaphysics itself, especially in its classical function as the inquiry into first principles and ultimate causes. As Lumbard (2025) argues, such frameworks reject ontological hierarchies while simultaneously instituting a covert hierarchy of functionality that privileges technoscientific methods and sidelines ethical, cultural, and spiritual dimensions of inquiry. MH thus promotes a pluriversal epistemology that reflects the layered complexity of human health, allowing diverse methodologies context-sensitive inquiry.

Bakar (2022) emphasizes that the human being is a unique microcosm (*al-ʿālam al-ṣaghīr*), integrating elements from all levels of cosmic reality—material, imaginal (*ʿālam al-mithāl*), and spiritual. At the center of this ontological structure lies the *qalb* (heart), which in Islamic metaphysics is not merely a physical organ but the seat of consciousness, moral discernment, and divine receptivity. Through *tazkiyah* (spiritual purification), the *qalb* cultivates virtues such as humility, patience, and compassion, which guide ethical action and support emotional and somatic balance (Yusuf, 2004). This resonates with the Prophetic teaching: “Truly, in the body there is a morsel of flesh which, if sound, the entire body is sound; and if corrupt, the entire body is corrupt. Truly, it is the heart” (*Ṣaḥīḥ al-Bukhārī*).

In this integrated view, virtuous living is not only health-promoting but also spiritually generative, attracting *barakah*—a form of divine grace that flows from ethical, intentional living and uplifts every dimension of human life. As Faris (2022) captures, “You’re conscious of what you eat and drink not because of physical health alone, but because what you eat impacts your energy and wellbeing, which ultimately impacts your journey to God.” This paradigm is especially crucial for understanding mental health and emotional-spiritual resilience, which have long resisted integration into mainstream healthcare models.

The MH framework emphasizes the centrality of virtue in healthcare, drawing on a multiplex conception of the human as a layered being—body, psyche, and spirit. This perspective aligns with Pellegrino’s (1985) vision of the physician as a moral agent and Vallor’s (2016) proposal for cultivating *technomoral virtues*—character traits such as compassion, prudence, and humility—needed to ethically navigate healthcare innovation in the technological age. Integrating virtue thus ensures healthcare attends holistically to individuals, harmonizing physical well-being with ethical integrity and spiritual flourishing.

This ontological view positions human beings as relational, embedded, and meaning-seeking agents whose well-being cannot be detached from moral purpose, spiritual orientation, and ecological attunement. In contrast to systems-based healthcare models that promise a *technical holism*—merely aggregating biological, behavioral, and environmental data—MH offers an integrative holism grounded in metaphysical, ethical, and epistemological plurality.

This is not to suggest that the MH framework moralizes illness or links it to moral failure. Health is understood as shaped by structural, relational, and spiritual factors, and must be assessed through empirical means. *Tazkiyah* is encouraged as a personal pursuit to strengthen one’s own moral and emotional resilience. In caring for others, however, the framework emphasizes *ḥusn al-ẓann* (having a charitable assumption about others)—withholding judgment and extending compassionate care using rigorous empirical methods.

### 2.2 Multiplex epistemology

Health is inherently complex, uncertain, and multi-dimensional, necessitating an epistemology that is equally layered and context-sensitive. The MH framework adopts a multiplex epistemology, which integrates empirical data, lived experience, clinical insight, and ethical reasoning, moving beyond traditional evidence hierarchies that prioritize randomized controlled trials (RCTs). This pluralistic approach is rooted in scientific rigor and aims to reflect the complexity of real-world health.

Drawing from Husserl’s phenomenology (as cited by Sturmberg, 2012), MH emphasizes that health cannot be fully understood without incorporating the first-person, lived experience of the patient. Polanyi (1966) complements this view with his concept of tacit knowledge and “knowing-how,” highlighting that much of what is essential to understanding health is embodied and cannot be fully articulated. Sturmberg and Morin (2008) further differentiate between knowledge as a “thing”—codified and structured data—and knowledge as a “flow,” which is adaptive, context-dependent, and embedded in practice. This view resonates with McGilchrist’s (2009) critique of modern science’s dominant, left-hemisphere–oriented epistemology, which marginalizes embodied intuition and moral imagination—both vital for holistic healing. While simplistic left/right brain dichotomies have been discredited, McGilchrist’s neurophilosophical synthesis offers a nuanced, evidence-based case for reintegrating complementary modes of knowing.

Aligned with Aristotle’s principle of appropriate precision, the MH framework acknowledges that different kinds of health-related questions call for different methodological approaches—be they quantitative, qualitative, interpretive, or normative—each offering its own form of precision and value depending on the context, purpose, and level of inquiry. For example, quantitative metrics may help identify population-level trends, but understanding psychosocial resilience or moral suffering demands idiographic, interpretive approaches. This flexibility allows MH to employ both nomothetic (generalizing) and idiographic (individualizing) perspectives as appropriate, reflecting the dynamic nature of health and the need for different types of knowledge.

While empirical tools are essential for measuring physiological variables, understanding suffering, healing, or ethical choice demands interpretive, experiential, and moral reasoning. MH incorporates Polanyi’s (1966) insight that personal, tacit knowing is central to practice, and Sturmberg’s (2012) argument that knowledge in healthcare must be both codified and emergent. To address complex questions, MH employs both triangulation and crystallization to integrate diverse epistemic sources. Triangulation links empirical data, lived experience, and normative reasoning—not to seek convergence, but to enrich understanding across domains. In line with post-positivist traditions, MH can also draw on “crystallization” (Richardson, 2000), which captures how complex realities refract through multiple, partial perspectives. Together, these approaches reflect MH’s commitment to epistemic humility, pluralism, and integrative inquiry.

MH modeling is inherently pluralistic, synthesizing mechanistic, statistical, and semiotic approaches. Page’s (2018) theory of many-model thinking and Helbing’s (2010) advocacy for pluralistic systems modeling converge here: no single model can capture the multifaceted realities of health. For instance, a mechanistic model might explain insulin resistance; a statistical model may predict diabetes onset; and a semiotic model could analyze the illness narratives surrounding food and identity. Gyllingberg et al. (2023) and Epstein (2008) caution against overfitting reality to abstract formalisms—a danger MH avoids by layering models rather than selecting one. The MH framework endorses both explanatory models (which seek causal understanding) and predictive models (which forecast future states), depending on context and need. While it is neither likely nor necessary for a single study to integrate all three dimensions comprehensively, MH encourages researchers to remain open to multiple perspectives and to pursue integration where contextually appropriate.

At the core of MH’s epistemology is triangulation, a method that integrates empirical data, qualitative narratives, clinical insights, and ethical reasoning, to enhance validity and deepen understanding. Rather than privileging either Big Data (aggregate datasets) or Small Data (individual-level insights like ethnographic or N-of-1 studies), MH uses triangulation to achieve consilience, or the convergence of evidence from independent sources—a hallmark of agile science (Hekler et al., 2016, 2019). By embracing a both-and logic (Smith and Lewis, 2022), MH resolves seeming dichotomies such as objectivity vs. subjectivity, science vs. spirituality, and prediction vs. understanding, not through compromise but through creative synthesis.

### 2.3 Multiplex methodology

The MH framework complements its ontology and epistemology with a multiplex methodology, designed specifically to address the complex, multi-dimensional realities of human health. In this section, we discuss two important aspects of multiplex methodology.

#### 2.3.1 Critical complexity and Open Science

Dominant biomedical paradigms in modern healthcare often strive for equilibrium and control, assuming predictability and stability as normative goals. Sturmberg (2013) critiques such approaches for oversimplifying the inherent complexity of living systems, thereby inadvertently limiting resilience and adaptability. In contrast, MH recognizes health systems as open-ended and dynamically evolving entities that resist full predictability or reductionist modeling. Acknowledging complexity implies not merely tolerating uncertainty but actively engaging it, incorporating multiple interpretations and decompositions of phenomena to better reflect the richness of real-world contexts.

In this regard, MH integrates insights from Critical Complexity—a perspective articulated by Cilliers (1998, 2016), Morin (2008), and further developed by Birhane (2021)—to address the inherent openness, dynamism, and relationality of health systems. Health, from this vantage point, is not reducible to a set of static physiological variables but emerges from continuously evolving interactions among biological, psychological, social, and environmental dimensions.

As AI becomes increasingly integrated into healthcare and other complex domains, it is essential to recognize that machine learning systems are neither epistemically neutral nor universally applicable. Dominant approaches often fall under what Birhane and Sumpter (2022) term Closed ML, which assume that complex, dynamic phenomena can be fully captured, optimized, and predicted through data alone. Such systems reflect a reductionist, Cartesian-Newtonian epistemology that privileges objectivity, control, and closure, often overlooking the historical, social, and ethical dimensions of the domains they are applied to. In contrast, our approach to applying AI in healthcare explicitly acknowledges that health systems are complex, dynamic, and value-laden. Rather than treating them as fully knowable or reducible to data-driven representations, we integrate human expertise, contextual understanding, and interpretive reasoning throughout the modeling process.

This positions our work closer to Open Machine Learning (Open ML), though in practice, elements of Partially Open ML are sometimes used when existing domain models are refined through data-driven methods. However, our commitment to maintaining openness to multiple perspectives, resisting premature closure, and embedding ethical and epistemic reflexivity aligns most directly with the principles of Open ML. As defined by Birhane and Sumpter, Open ML recognizes the inherent indeterminacy and situatedness of complex systems, employs a plurality of models—statistical, narrative, and qualitative—and centers human judgment and values, making it especially appropriate for ethically and socially embedded fields such as healthcare.

This vision also aligns with Senturk’s (2011) concept of *Open Science*, which offers a structural alternative to the reductionist tendencies of modern “closed science.” While closed science is grounded in a unilayered ontology, singular epistemology, and methodological monism, Open Science embraces *multiplex ontology*, *multiplex epistemology*, and *methodological pluralism*. It recognizes that scientific knowledge is not absolute or static but partial, evolving, and deeply shaped by historical and cultural context. Rather than imposing a singular view of truth, Open Science affirms the coexistence of multiple valid approaches to understanding reality. This structural shift enables science to better engage with complexity, support open societies, and prevent exclusionary and authoritarian attitudes.

#### 2.3.2 Comparative multiplex ethics through *BioFiqh*

A key innovation of the MH framework is *BioFiqh*—a multi-level normative approach rooted in *uṣūl al-fiqh* that enables rigorous, context-sensitive ethical medical decision-making under uncertainty. Islamic jurisprudence (*uṣūl al-fiqh*) is a science of ethical and legal reasoning that derives judgments from revelation to guide human action. As Senturk et al. (2020) notes, fiqh historically fulfilled many of the functions later claimed by Western social science—interpreting norms, structuring society, and shaping moral life. Its layered classification of norms exemplifies a non-binary, multiplex model of ethical reasoning. This highlights a key insight of the MH framework: i.e., decolonial, pluralistic approaches to social science and ethics are not only possible, but necessary for transcending Eurocentric and overly technocratic paradigms in healthcare. MH enables EBM with multi-layered moral reasoning grounded in a rich tradition of virtue ethics, legal epistemology, and spiritual insight.

**Figure 2:**
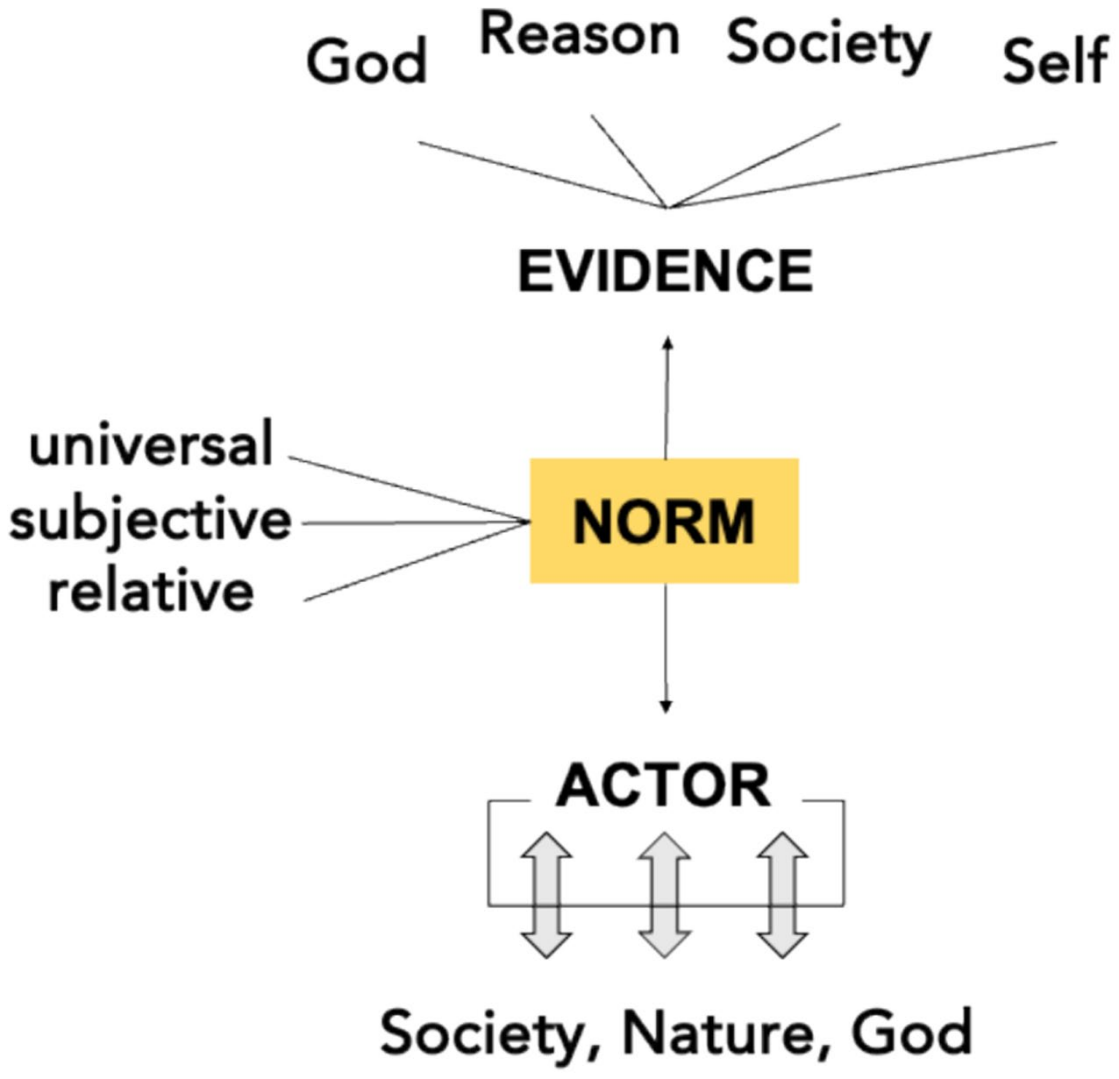
A multi-layered ethical reasoning framework based on Islamic jurisprudence (uṣūl al-fiqh), distinguishing universal (qaṭʿiyyāt), relative (ẓanniyyāt), and subjective (mubāḥāt) norms—enabling principled moral reasoning under uncertainty.

Figure 2 illustrates MH’s layered approach to moral reasoning. By distinguishing multiple levels degrees of certainty and normativity, MH challenges the binary logic characteristic of currently dominant ethical frameworks, which often prioritize universal, decontextualized moral rules. MH exemplifies how normative reasoning can be multi-layered, plural, and context-sensitive, offering principled flexibility suited to complex clinical decision-making.

At its core, *BioFiqh* is characterized by a *multiplex structure of normativity* that distinguishes between three domains of ethical reasoning (Senturk, 2022):

- *Qaṭʿiyyāt (Definitive/Universal Norms):* Rooted in certain textual or scientific evidence, these norms command obligatory action or abstention.
- *Ẓanniyyāt (Probable/Relative Norms):* These arise from non-definitive, interpretive evidence and are recommended or discouraged depending on context.
- *Mubāḥāt (Permissible/Subjective Norms):* These pertain to morally neutral choices, left to individual discretion and circumstance.

In a healthcare context, universal norms may govern non-negotiable ethical obligations, such as preserving life or prohibiting direct harm, while relative norms can guide context-dependent issues—for example, in decisions around end-of-life care or mental health interventions—where interpretive judgment is needed. Permissible or discretionary norms allow room for individual preferences and cultural variation such as dietary choices, modesty practices, or complementary therapies.

Critically, *BioFiqh* also emphasizes evidentiary integration, drawing simultaneously from two epistemic sources: (1) *fiqh-based* ethical rulings, which are grounded in scriptural texts and centuries of legal deliberation, and (2) *scientific-medical* evidence, which offers empirical insight into physiological and psychosocial outcomes. While BioFiqh is grounded in Islamic jurisprudence and offers a valuable example for Muslim contexts, the broader MH framework is inherently pluriversal. It is designed to flexibly accommodate diverse normative traditions—religious or secular—based on the sociocultural and epistemic commitments of the communities in which it is engaged. This combination creates a framework where normative judgments are both ethically robust and empirically responsive. Medical *fatwās*, for example, are often issued through this integrative process, assessing both the scientific validity of an intervention and its moral implications considering Islamic principles.

This tripartite classification allows MH to accommodate *varying degrees of epistemic certainty* without defaulting to moral paralysis. In most real-world medical situations, definitive knowledge is often elusive. *Uṣūl al-fiqh* embraces this reality and considers probabilistic, uncertain evidence (*ẓann*) as sufficient for moral reasoning and action—making it uniquely suited for contemporary healthcare, where real-world evidence in the form of personalized medicine (N-of-1 trials) and patient narratives often challenge rigid evidence hierarchies.

## 3 Case study: MH-based population health management

To illustrate the applied value of the MH framework, we examine its integration into Population Health Management (PHM)—a multidisciplinary field that unites clinical care, public health, informatics, and behavioral science. While PHM already emphasizes longitudinal health trajectories, upstream prevention, and system-wide coordination, MH offers a complementary ethical-epistemological lens that foregrounds pluralism, normative inclusivity, and responsiveness to cultural and spiritual dimensions—especially vital for ethically diverse, global contexts.

As Valles (2020) argues, Population Health science emerged to counter the limitations of biomedical individualism by emphasizing the social embeddedness of health and the need for intersectoral collaboration. PHM operationalizes this through AI-enabled risk stratification, EHR-integrated care pathways, and lifestyle-oriented interventions (Nash et al., 2021; Steenkamer et al., 2016). Yet while PHM provides the infrastructure for systemic, data-driven interventions, MH deepens its conceptual and moral scope—supporting the inclusion of multiple worldviews, ethical traditions, and non-biomedical indicators of well-being.

Building on this, Henry (2025a, 2025b) proposes a comprehensive model for embedding the Human Phenotype Ontology within Population Health Management, proposing federated infrastructures such as Higher Expert Medical Science Safety (HEMSS), predictive pre-eXams, and AI-powered biological modelling as cornerstones for ethically robust, equitable care delivery. While Henry’s approach ensures structural readiness and policy alignment, the MH framework adds layers of interpretive nuance: integrating community-derived narratives, cultural-spiritual insights, and pluralistic moral frameworks (e.g., BioFiqh, virtue ethics) making PHM not just technologically capable, but deeply humane and contextually responsive.

In the adolescent mental health use case, this means that predictive alerts generated through HPO–Biological Modelling can be enriched with relational assessments of spiritual distress, family norms, or communal meaning-making—ensuring that risk stratification is not only precise but also person-centered. This synthesis maintains infrastructural integrity while elevating ethical resilience and global cultural responsiveness.

The MH framework offers a principled enhancement to PHM by introducing six interlocking contributions. It expands health beyond the physical to include psychological, social, and spiritual dimensions (*multiplex ontology*), and integrates ethical, narrative, and experiential knowledge alongside clinical data (*multiplex epistemology*). It incorporates diverse modeling approaches—mechanistic, statistical, symbolic, and normative—to reflect the full complexity of health systems (*pluralistic modeling*). MH embraces contextual variability and emergent phenomena (*critical complexity*), balances big data with clinical insight and moral reasoning (*triangulated science*), and brings culturally grounded ethical frameworks such as BioFiqh and virtue ethics to bear on issues like adolescent autonomy and care (*comparative multiplex ethics*). While a full operational model lies beyond this paper’s scope, these principles sketch how MH can help PHM evolve into a more ethically resilient, culturally sensitive, and holistically grounded paradigm.

As a concrete example, consider an adolescent mental health initiative embedded within a PHM framework that uses HPO, predictive AI, and federated learning to identify early markers of depression and anxiety. While such technologies offer scalable risk stratification, they often overlook interpretive depth and cultural attunement. For instance, an adolescent flagged as high risk may be experiencing spiritual distress or family-based moral tension that is invisible to algorithmic models. Without broader frameworks, interventions may become overly biomedical and insufficiently person-centered.

This is where MH becomes indispensable, PHM already advances a life course orientation by tracking health trajectories across time and contexts; MH can further enhance this by embedding those trajectories within dynamic social, ethical, and spiritual worlds. Where PHM and Life Course Theory (LCT) emphasize developmental timing and social determinants, MH brings in the pluralistic meanings that individuals and communities assign to those life transitions. A genomic risk flag for adolescent depression, for instance, may indicate more than biochemical vulnerability—it may reflect spiritual despondency, strained familial ethics, or cultural tensions.

MH enables these nuances to surface by integrating narrative insight, value-laden judgment, and community-based reasoning alongside data analytics. In doing so, it supports a more contextual and relational understanding of health that aligns closely with LCT’s emphasis on time, place, and cumulative experience. Rather than imposing uniform interventions, MH fosters adaptive, culturally resonant responses that respect autonomy while honoring communal norms. This synthesis of predictive modeling with moral meaning-making allows PHM to evolve into a more humane, just, and globally relevant practice.

## 4 Discussion: How MH framework advances medicine and healthcare

The MH framework offers more than a philosophical realignment—it proposes a rigorous, pluralistic, and actionable scientific paradigm grounded in critical complexity, multi-layered normativity, and open science. In contrast to dominant health frameworks built on predictive modeling, linear causality, and narrow epistemologies, MH fosters a shift toward understanding, context, and meaningful relational engagement. MH presents an example of a pluralistic open science that does not reject of scientific rigor, but reimagines what rigorous science would mean when we assume multiplex ontology and epistemology, which is indispensable for the study of living systems like human health.

As Juarrero (1999) and Morin (2008) argue, living systems are not reducible to mechanistic parts but are dynamic, non-linear, and emergent. Health systems, in particular, are shaped by feedback loops, cultural beliefs, and individual experiences. The overreliance on closed, predictive logic can result in active harm, especially to the people who are already disadvantaged in the society as Birhane (2021) demonstrates. Closed ML systems that operate on static datasets and fixed models are ill-equipped to handle the context-sensitive and evolving realities of health, especially when these tools exacerbate inequalities among already marginalized populations.

The closed worldview of predictive optimization seeks to eliminate uncertainty, but in doing so it erodes human agency, pluralism, and ethical reflection. The MH framework, by contrast, advances a both-and approach that embraces uncertainty and integrates predictive science with interpretive understanding. This paradigm resonates with E. F. Schumacher’s call for “wisdom-oriented science”—a science that does not just explain and predict, but also nurtures meaning, responsibility, and the common good. MH follows this call by integrating semiotics, spirituality, and ethics directly into the scientific method and provides a conceptual foundation for achieving this integration.

MH also grounds healing as becoming whole, returning to the etymological roots of health from the Old English hal—meaning wholeness. As Sturmberg (2012) notes, “wholeness is experiential,” and only the patient can truly judge if it has been achieved. The WHO (1946) definition of health—emphasizing complete physical, mental, and social well-being—recognizes this multidimensionality but has rarely been fully operationalized in modern systems (Sturmberg, 2012). MH takes this challenge seriously, integrating spiritual flourishing, virtue ethics, and moral reasoning into the pursuit of health outcomes.

- **Virtue Ethics and Tazkiyah (Spiritual Purification)**

MH’s inclusion of Islamic virtue ethics such as *tazkiyah* offers a normative anchor for behavioral change strategies within PHM. These concepts are not merely theological abstractions—they provide culturally resonant motivations for practices like healthy eating, sleep hygiene, and emotional self-regulation. By drawing on virtues like *sabr* (patience), *hikmah* (wisdom), and *tawakkul* (trust in God), MH reinforces PHM’s emphasis on preventive care and mental well-being while ensuring that behavioral interventions are rooted in indigenous moral vocabularies. In doing so, MH makes behavioral science in PHM both more effective and more ethically grounded.

- **Communal Support and Social Responsibility (*Ummah*)**

The MH framework also strengthens PHM by foregrounding community (*ummah*) as both an ontological reality and a practical structure for care. For instance, rather than viewing mental health as an individual burden, MH encourages shared responsibility for well-being through culturally meaningful communal spaces—mosques, local health councils, neighborhood support networks. This communal logic can inform PHM interventions such as peer mentoring, communal fasting support, or collective parenting education. In contexts where individualism dominates biomedical assumptions, MH offers a corrective grounded in relational ethics and mutual accountability.

- **Informed Choice and Autonomy through BioFiqh**

Finally, MH’s integration of *BioFiqh* enables PHM systems to navigate the fine balance between predictive analytics and respect for personal agency. Through its ethical scaffolding—*Qaṭʿiyyāt* (definitive rulings), *Ẓanniyyāt* (probabilistic judgments), and *Mubāḥāt* (permissibles)—BioFiqh guides PHM in deploying nudges and predictive tools in a way that values religious rituals and population dignity. For example, in the case of mental health, overreach can quickly become coercive. MH ensures that ethical deliberation is not an afterthought but built into the epistemic architecture of PHM interventions.

At the civilizational level, MH advances a vision of open science as a precondition for an open civilization. Senturk (2011, 2025) and Birhane (2021) argue that true openness in science requires more than data transparency—it requires openness to multiple ways of knowing, to relational thinking, and to the coexistence of different normative systems. This is especially critical in plural societies where health must be negotiated across traditions. As Qadir and Senturk (2024) note, the emergence of Multiplex AI Humanities is part of this broader shift—a movement toward systems of knowledge that are ethical, interpretive, and human-centered.

Ultimately, MH is better science precisely because it acknowledges the complexity, diversity, and meaning-laden nature of human lives and health. It integrates what is measurable with what is meaningful; it values prediction, but not at the expense of understanding. It provides a model of care and knowledge that is as rigorous as it is relational, as inclusive as it is integrative. In doing so, it opens a path not only to better health outcomes but also to a wiser, more just, and more pluralistic scientific and civilizational future.

## 5 Conclusion

The Multiplex Health (MH) framework introduces a normative and conceptual scaffolding that is urgently needed in the evolving landscape of healthcare. Beyond its philosophical contributions, MH offers actionable pathways for advancing Population Health Management (PHM), particularly in addressing mental health and behavioral challenges. The case study presented in this paper demonstrates how MH can inform a more context-aware and ethically robust approach to PHM—one that respects individual agency, cultural diversity, and the layered nature of human well-being. In sum, the MH framework as applied to PHM exemplifies how a complexity- and normativity-informed paradigm can elevate public health from a system of management to a system of meaning. This integrative vision holds promise for building a more holistic, open, and pluralistic healthcare system capable of addressing the moral and practical challenges of our time.

## Data Availability

The original contributions presented in the study are included in the article/supplementary material, further inquiries can be directed to the corresponding author.
